# Cardiac tamponade developing during Trousseau's syndrome with pulmonary embolism

**DOI:** 10.1186/s40981-023-00678-w

**Published:** 2023-12-01

**Authors:** Yuya Itakura, Takahiro Hakozaki, Satoki Inoue

**Affiliations:** Division of Intensive Care, Fukushima Medical University Hospital, 1 Hikarigaoka, Fukushima City, Fukushima 960-1295 Japan

To the Editor

Cardiac tamponade and pulmonary embolism are known complications of malignancy, but it's exceptionally rare for both conditions to occur simultaneously. We present a case where cardiac tamponade developed alongside pulmonary embolism during Trousseau's syndrome.

A 42-year-old woman was admitted to our hospital experiencing severe dyspnea and tachycardia. She had a history of lung adenocarcinoma and was currently undergoing anticoagulant treatment for pulmonary embolism related to Trousseau's syndrome (Fig. 1-A). Her pulmonary embolism was significant, with an echocardiographic exam showing a transtricuspid pressure gradient of 66 mmHg. Due to her advanced stage, she only received anticoagulant therapy, already prescribed for Trousseau's syndrome. After admission, emergency echocardiography revealed a substantial pericardial effusion, although both cardiac chambers were maintained with reduced volumes (Fig. 1-B). Her blood pressure (BP), heart rate (HR), and respiratory rate (RR) were 140/50 mmHg, 120 bpm, and 30 bpm, respectively.

**Fig. 1:**
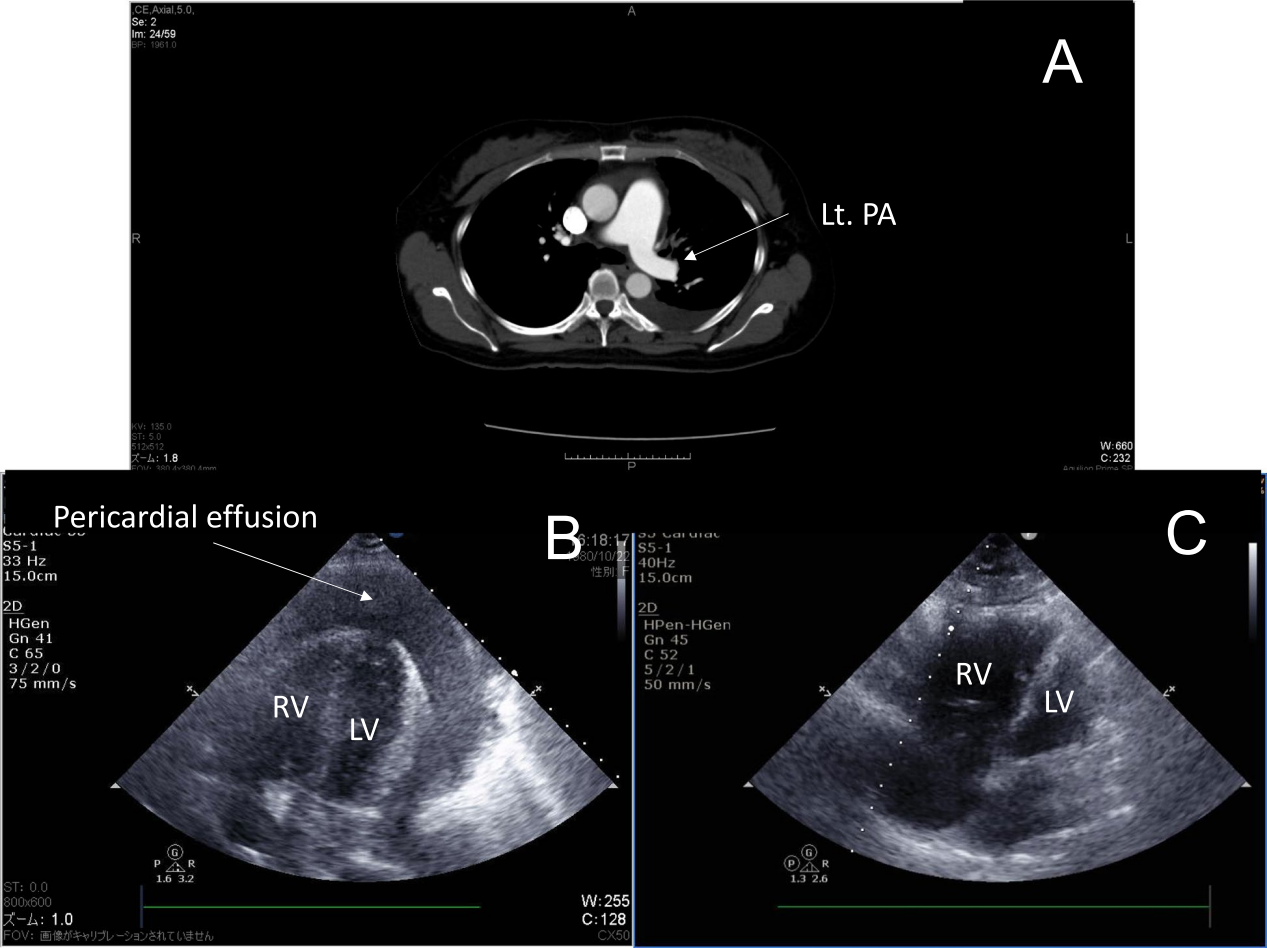
Images of CT pulmonary angiogram and transthoracic echocardiography. **A** CT pulmonary angiography demonstrates pulmonary embolism in the left main pulmonary artery (arrow head) a day before pericardiocentesis. **B** and **C** Apical four chamber view on transthoracic echocardiography. Massive pericardial effusion before pericardiocentesis (**B**); remarkably dilated right ventricle and decompressed left ventricle after removal of pericardial effusion (**C**). Lt.PA; left main pulmonary artery, RV; right ventricle, LV; left ventricle

Pericardiocentesis was performed after stopping anticoagulant therapy, and 500 ml of bloody fluid was drained. However, her symptoms did not improve. Over the next few hours, her condition worsened, and echocardiography showed minimal pericardial effusion but the characteristic D-shape of the right ventricle (RV) and the decompressed left ventricle (LV) (Fig. 1-C). Although her BP was stable, HR and RR increased to 140 bpm and 40 bpm, respectively. Nasal high-flow oxygen therapy was administered for palliative care.

Managing both cardiac tamponade and pulmonary embolism is challenging due to the conflicting use of anticoagulants needed for pulmonary embolism and the relative contraindication of anticoagulants in bloody pericardial effusion. Pericardiocentesis, the primary treatment for cardiac tamponade, poses a dilemma, as it can increase right ventricle (RV) filling by draining fluid causing tamponade, potentially worsening left ventricle (LV) compression when pulmonary embolism (PE) is present [1]. Our case, and others, have reported deteriorated conditions post-pericardiocentesis, possibly due to similar mechanisms [1, 2]. Although the coexistence of PE was not recognized in a prior report, it's known that concurrent PE can maintain right heart filling even with massive pericardial effusion, preventing excessive right heart dilatation induced by PE and maintaining systemic hemodynamics [3].

The decision to perform pericardiocentesis becomes a clinical dilemma. While some cases have reported immediate hemodynamic improvement after pericardiocentesis despite concurrent PE [4], our case presented a challenge. We opted to take a risk, recognizing that the improvement in hemodynamics after pericardiocentesis might hinge on the delicate balance between the degree of PE and pericardial effusion. The reason we took a risk in this case was because her family strongly wished to prevent her inevitable gradual decline through mere observation, which naturally necessitated obtaining sufficient informed consent from both the patient and her family.

## Data Availability

Not applicable.

## Abbreviations

PE: Pulmonary embolism
BP: Blood pressure
HR: Heart rate
RR: Respiratory rate
RV: Right ventricle
LV: Left ventricle
